# Genome-Wide Analysis of Soybean Polyamine Oxidase Genes Reveals Their Roles in Flower Development and Response to Abiotic Stress

**DOI:** 10.3390/plants14121867

**Published:** 2025-06-18

**Authors:** Yang Yu, Bohuai Jin, Meina Gao, Ke Zhang, Zhouli Liu, Xiangbo Duan

**Affiliations:** 1 College of Life Science and Engineering, Shenyang University, Shenyang 110044, China; yuyang_syu@163.com (Y.Y.); zlliu@syu.edu.cn (Z.L.); 2 Key Laboratory of Black Soil Evolution and Ecological Effect, Ministry of Natural Resources, Shenyang 110000, China; 3 Liaoning Key Laboratory of Urban Integrated Pest Management and Ecological Security, Shenyang 110044, China; 4 Northeast Geological S&T Innovation Center of China Geological Survey, Shenyang 110000, China

**Keywords:** *Glycine max* L., PAO, bioinformatic analysis, tissue-specific expression, stress response

## Abstract

Polyamine oxidase (PAO) is an important enzyme that functions in the catabolism of polyamines. While plant PAOs have been studied in several species, there is a lack of research on this gene family in soybean (*Glycine max* L.), one of the major food crops worldwide. Here, a genome-wide analysis identified 16 *GmPAOs* from the soybean genome, which were unevenly distributed in nine soybean chromosomes and were then phylogenetically classified into three groups. Collinearity analysis identified 17 duplicated gene pairs from the *GmPAO* family, and their Ka/Ks values were all less than one, indicating that the *GmPAO* family has undergone purifying selection during evolution. Analyses of the conserved motif and gene structure revealed the sequence differences among the GmPAOs of the three groups, suggestive of their functional differentiation. Additionally, the prediction of the secondary and tertiary structure of the GmPAOs provided a further basis for revealing their biological functions. A number of *cis*-acting elements relevant to development, phytohormone, and stress response were discovered in the promoter regions of the *GmPAOs*, which might be responsible for their functional diversities. Expression pattern analysis indicated that more than half of the *GmPAOs* showed preference in flower, two showed specificity in stem and shoot apical meristem, whereas four were barely expressed in all samples. Expression profiling of the *GmPAOs* also revealed that they were involved in the response to abiotic stresses, including cold, drought, and especially submergence stress. All these results lay an important foundation for further characterizing the functional roles of *GmPAOs* in soybean development and response to abiotic stresses.

## 1. Introduction

Polyamines (PAs) are small aliphatic amines with high biological activity found in almost all living cells. In plants, putrescine (Put), spermidine (Spd), spermine (Spm), and thermospermine (Tspm), an isomer of Spm, are the major PAs, owing to their presence in most lineages [1,2], which are involved in multiple aspects of growth and development processes, like embryogenesis [3], leaf morphology [4], vein definition [5], flowering [6], seed germination [7], senescence [8], and so on.

A series of anabolic and catabolic enzymes are implicated in the metabolic routes of PAs, and they corporately determine the in vivo PA homeostasis [9]. Two classes of amine oxidases mainly account for the catabolism of PAs, namely diamine oxidases (DAOs) and polyamine oxidases (PAOs). The former catalyze the oxidation of Put to generate 4-aminobutanal, H_2_O_2_, and ammonia, while in contrast, the latter use Spd, Spm, or Tspm as substrates [1,10].

PAOs bear a non-covalently bound molecule of flavin adenine dinucleotide (FAD) and rely on FAD for their function [2,9]. According to the differentiation in terms of reaction mode, substrates, and products, plant PAOs are categorized into two groups. One is involved in PA terminal catabolism (TC), producing 1,3-diaminopropane (DAP), H_2_O_2_, and the respective aldehydes. Typical examples are maize ZmPAO1, barley HvPAO1 and HvPAO2, and rice OsPAO7, all of which are TC-type enzymes that prefer Spm and Spd as substrates [11]. In turn, more PAOs belong to the other group that functions in the back-conversion (BC) reactions of PAs, oxidizing Spm/Tspm to Spd and/or Spd to Put, with the concomitant production of 3-aminopropanal and H_2_O_2_ [12,13]. For example, all AtPAOs from *Arabidopsis thaliana* are classified into the BC-type group, and their catalytic properties have been experimentally verified in previous studies [14,15,16].

Functional characterization of *PAOs* regarding plant growth and development has been performed in various species. In *Arabidopsis*, AtPAO5 functions in the elongation of stem and root by regulating xylem differentiation. The loss-of-function mutants produced longer stems and roots than the wild type, while the overexpressing line exhibited a semi-dwarf phenotype and presented shorter roots [17]. Furthermore, a recent study also revealed AtPAO5’s role in shoot meristem formation [18]. Similarly, using the gene knockout mutants and overexpressors, AtPAO2 was found to contribute to seed germination, early seedling development, root morphogenesis, and ABA-mediated vegetative growth [19,20]. In addition to the model plant *Arabidopsis*, researchers also studied the function of PAOs in crop plants. For instance, the maize (*Zea mays*) PAOs were reported to have a role in light- and auxin-mediated differentiation of the cell wall in mesocotyl epidermal tissues [21]. The rice (*Oryza sativa*) genome includes seven PAO-encoding genes, and three of them (*OsPAO3*, *4*, and *5*) are the most abundantly expressed in seedlings and mature plants [22]. *OsPAO5* negatively regulates mesocotyl elongation, and targeted mutagenesis of the gene also remarkably increased grain weight, grain number, and yield, shedding light on the application potential of plant PAOs in agricultural breeding [23]. However, OsPAO7, the TC-type enzyme, is specifically expressed in rice anther, where it was hypothesized to deliver H_2_O_2_ for secondary wall thickening through lignin formation [11]. Additionally, the involvements of PAOs in programmed cell death (PCD) were also previously discovered [24,25].

The functional role of plant PAOs is not restricted to participating in regular growth and development but also embodied in regulating the response to external stressors. Different maize *PAOs* were responsive to heat, drought, and salinity stress. Functional analysis indicated that *ZmPAO6* enhanced heat tolerance through mediating polyamine catabolism when heterogeneously expressed in *Arabidopsis* [12]. The pepper (*Capsicum annuum*) *CaPAO2* and *CaPAO4* were induced by cold treatment, and their overexpression in *Arabidopsis* improved freezing tolerance [26]. In rice, salt stress elevated the expression level of *OsPAO3*, which could boost plant salt tolerance at the germination stage [27]. On the other hand, plant PAOs might also negatively regulate stress resistance. For example, knockout of *AtPAO5* increased salt tolerance through Tspm-triggered metabolic and transcriptional reprogramming, while overexpression of the sweet orange (*Citrus sinensis*) *PAO4* inhibited both vegetative growth and root elongation under salt stress [28,29]. Moreover, it was recently reported that mutation of tomato (*Solanum lycopersicum*) *SlPAO3* enhanced drought tolerance due to diminished xylem hydraulic conductivity [30].

The *PAO* gene family has been identified in crop plants like rice [23], maize [12], pepper [26], cotton (*Gossypium hirsutum*) [31], sweet orange [32], and *Camellia sinensis* [33]. However, in soybean, the leading source of plant protein and edible oil worldwide, although several *PAO* homologs have been cloned and functionally analyzed [34,35], the biochemical characteristics and functions of the whole family are still poorly understood. In the present study, we identified 16 *GmPAOs* from the soybean genome and conducted a systematic bioinformatics investigation on this gene family. Then, the expression pattern of the *GmPAOs* in various organs/tissues was examined. Additionally, based on the transcriptome data, we also investigated the expression profiles of the *GmPAOs* in response to a number of abiotic stress conditions (salt, heat, cadmium, cold, drought, and submergence) and found that they were mainly responsive to cold, drought, and submergence stress. Our work lays an important foundation for further functional characterization of soybean *PAO* genes.

## 2. Results

### 2.1. Identification and Characterization of Soybean PAO Family Genes

Through a BLASTP search and further screening, a total of 16 polyamine oxidase encoding genes were identified from the soybean genome, which were named *GmPAO1*–*GmPAO16* according to their ID order (Table 1). The gene length of the *GmPAOs* ranged from 1617 (*GmPAO8*) to 9057 bp (*GmPAO13*), while their protein length varied from 385 (GmPAO1) to 600 aa (GmPAO5). The physicochemical parameters of the GmPAOs were calculated to obtain a preliminary understanding of these proteins (Table 1). The results indicated that GmPAO1 holds the minimum MW (43.29 kDa), while the maximum belongs to GmPAO5 (65.91 kDa). The pI of all the GmPAOs was less than 7, spanning from 5.00 (GmPAO7) to 6.06 (GmPAO8). For the instability index, those of eleven GmPAOs were below 40, suggestive of their stable nature; the other GmPAOs were classified as unstable. Fifteen GmPAOs were predicted as hydrophilic proteins with negative GRAVY values; only GmPAO16 has a positive GRAVY value and, thus, was considered to be hydrophobic.

### 2.2. Chromosomal Mapping and Phylogenetic Analysis of GmPAOs

Chromosomal localization analysis showed that the 16 *GmPAOs* were unevenly distributed in nine soybean chromosomes (Figure S1). Chr02 contains three *GmPAOs*, followed by Chr09, 13, 14, 15, and 18, all containing two, while Chr08, 10, and 17 each harbored only one *GmPAO* gene.

**Figure 1:**
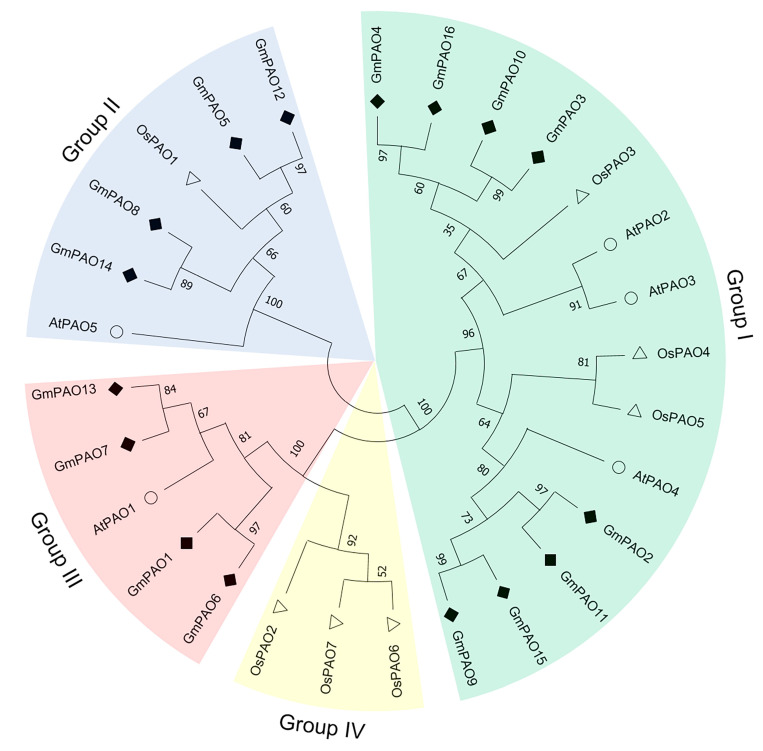
Phylogenetic relationship of polyamine oxidases from *Arabidopsis*, rice, and soybean. The evolutionary tree was constructed by MEGA-X software through the maximum likelihood (ML) method and Le_Gascuel_2008 model. Bootstrap analysis (1000 replicates) was conducted to estimate the reliability of the tree. The four groups are distinguished by different color areas. PAO proteins of *Arabidopsis*, rice, and soybean are marked with an empty circle, an empty triangle, and a solid square, respectively.

The amino acids of *Arabidopsis*, rice, and soybean PAOs were used to construct a phylogenetic tree to analyze their evolutionary relationships. These proteins were classified into four groups, and the number of members varied among them (Figure 1). Group I was the largest one, containing three *Arabidopsis*, three rice, and eight soybean PAOs; the next was group II, having one *Arabidopsis*, one rice, and four soybean members. Notably, for group III and IV, the former only includes PAO proteins from *Arabidopsis* (1) and soybean (4), while the latter only includes three rice PAOs.

### 2.3. Collinearity Relationship and Evolutionary Constraint of the GmPAO Family

**Figure 2:**
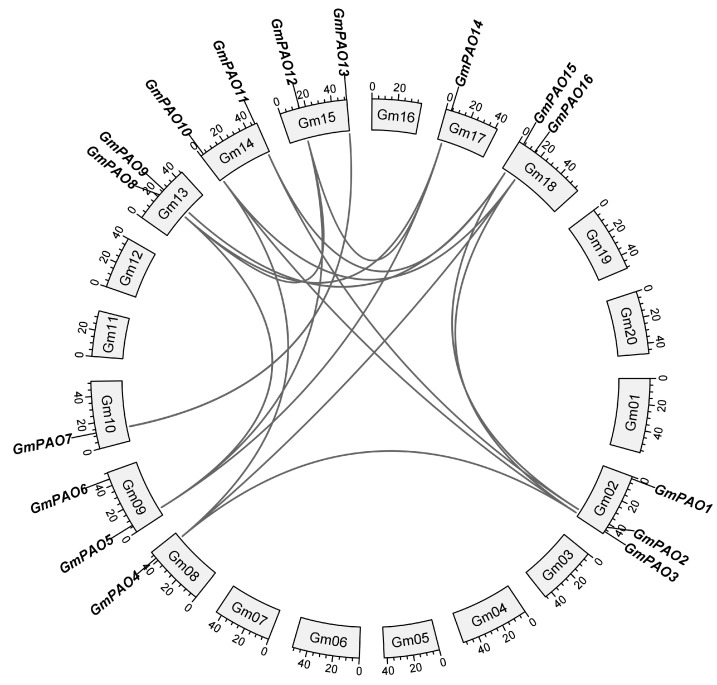
Collinear analysis of *GmPAOs*. The 20 soybean chromosomes are situated in a circle, and *GmPAOs* are mapped to them. Collinear gene pairs are linked using gray curves.

Collinearity analysis was performed to better understand the interrelation of the soybean *PAOs*. We identified 17 duplicated gene pairs from the *GmPAO* family (Figure 2). Specifically speaking, 10 of the duplication events occurred within genes from group I, and group II *GmPAOs* make up six gene pairs, while group III only contains one gene pair, with *GmPAO1* and *GmPAO6* not included in the duplication events. The gene duplication not only significantly drove the expansion of the *GmPAO* family but also contributed to the functional diversification of different *GmPAO* genes.

The Ka/Ks values of the duplicated gene pairs were calculated to assess the selective pressure during evolution. It was found that all Ka/Ks ratios were less than 1, ranging from 0.15 to 0.34 (Table S1), suggesting that the soybean *PAO* gene family has undergone strong purifying selection. Based on the Ks values, the divergence times of the *GmPAO* gene pairs were computed. More than half of the duplication events occurred around 40 mya, with the earliest being 48.25 mya, while the latest gene duplication occurred about 7.56 mya.

### 2.4. Conserved Motif and Gene Structure Analyses

**Figure 3:**
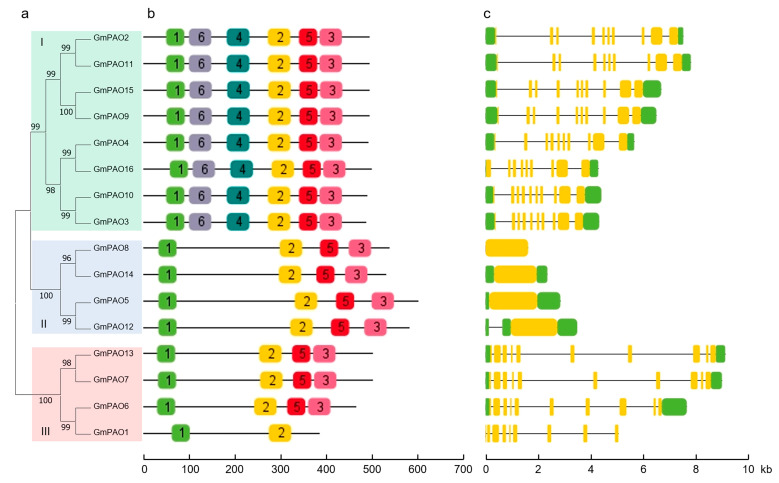
Phylogenetic relationship, conserved motif, and gene structure of GmPAOs. (**a**) Phylogenetic tree constructed with the amino acid sequences of GmPAOs by the ML method and bootstrap analysis (1000 replicates). The three GmPAO groups (I–III) were indicated with different color areas. (**b**) Conserved motif analysis. The 6 motifs are denoted with different colored rectangles. (**c**) Exon–intron structure of *GmPAO* genes. Green rectangles indicate UTRs, yellow rectangles indicate exons, and black lines indicate introns.

MEME suite was employed to investigate the conserved motifs of the GmPAOs, and six were finally identified (Figure S2). Motifs 1, 2, 5, and 3 were distributed in fifteen out of the sixteen GmPAOs and, thus, were considered as characteristic motifs for the family (Figure 3a,b). Group I GmPAOs appear quite different from those of the other two groups, as they include all the six conserved motifs, contrasting sharply with the other GmPAOs that only include the four characteristic motifs. GmPAO1 has the smallest protein length and possesses only motifs 1 and 2 in its sequence.

In terms of gene structure, group II *GmPAOs*, which all contain a single exon, are clearly distinguishable from those of the other two groups (Figure 3a,c). As for group I and III, although all *GmPAOs* from both groups are composed of nine or ten exons, genes in the same group resemble each other more in exon length and distribution. Analyses of the conserved motifs and gene structure are indicative of the functional differentiation among the groups and similarities within the group members.

### 2.5. Modeling of the Secondary and Tertiary Structures of GmPAOs

The secondary structures of the GmPAOs were analyzed with the SOPMA database (Table S2). Random coil accounted for the highest proportion (42.37–51.46%), followed by α-helix (31.69–39.96%), and the proportion of extended strand was the lowest (14.63–20.00%) for all GmPAOs. A similar structural composition indicated that these proteins might form similar higher-order structures. Therefore, we further predicted the 3D structures of the GmPAOs using the Alphafold server. Corresponding with the secondary structure analysis, all the GmPAOs are mainly composed of random coil, α-helix, and extended strand (Figure S3). In addition, although different GmPAOs displayed differences in tertiary structures to some extent, those phylogenetically close proteins tend to have more similar structures, like GmPAO2/GmPAO11 and GmPAO8/GmPAO14, indicative of their functional conservation.

### 2.6. Analysis of GmPAO Promoters

**Figure 4:**
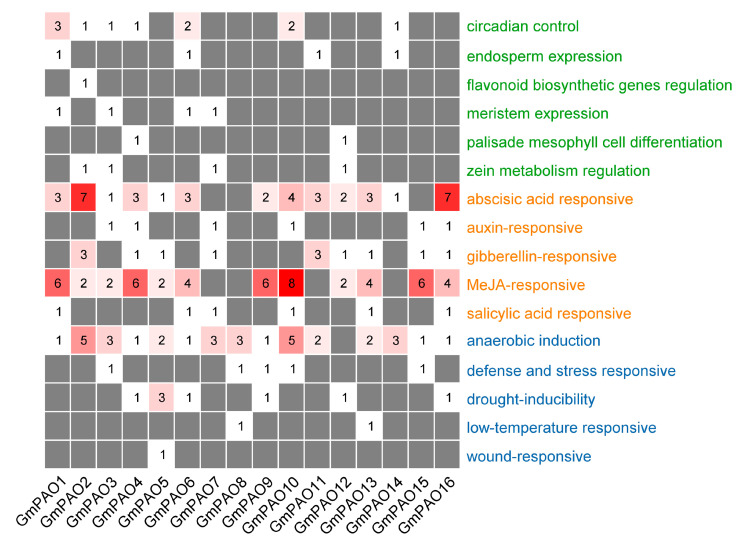
Promoter analysis of *GmPAOs*. *Cis*-acting elements related to development (green), plant hormone response (orange), and stress response (blue) were identified in the promoters of *GmPAOs*. The numbers of each kind of element are shown in separate color blocks.

The *cis*-acting elements in the promoter region of the *GmPAOs* were investigated to explore the regulatory pathways they participate in. We observed many development-, plant hormone-, and stress-related elements in the promoters of the *GmPAOs* (Table S3). Regarding the development-related elements, those responsible for circadian control were the most abundant, mainly distributed in the promoters of *GmPAO1*, *6*, and *9*. In addition, the elements involved in endosperm expression, flavonoid biosynthesis, meristem expression, palisade mesophyll cell differentiation, and zein metabolism were also identified in the promoters of various *GmPAOs* (Figure 4). Abscisic acid- and MeJA-responsive elements were the top two enriched plant hormone-related elements, which were found in almost all *GmPAO* genes, followed by gibberellin-, auxin-, and salicylic acid-responsive elements, consecutively (Figure 4). In the stress-related category, elements involved in anaerobic induction were discovered in all *GmPAOs* except *GmPAO12*. Eight drought-inducibility elements were found in six *GmPAOs*, and *GmPAO5* possesses three of them. Other elements in this category include those responsible for responsiveness to defense and stress, low temperature, and wound (Figure 4). The diversity of *cis*-acting elements in promoter regions might contribute to the functional diversity of *GmPAOs* in regulating various physiological and metabolic pathways.

### 2.7. Expression Pattern of GmPAOs in Different Soybean Organs/Tissues

**Figure 5:**
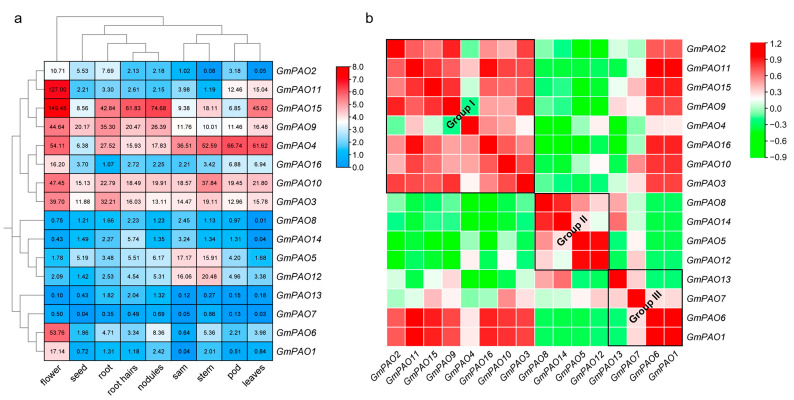
Expression analysis of *GmPAOs* in different soybean organs/tissues. (**a**) Expression level of *GmPAOs* in nine soybean organs/tissues; numbers in the color blocks indicate FPKM values. The color scale represents log2 format-converted FPKM values, with red indicating high expression levels and blue indicating low expression levels. (**b**) Expression correlation of *GmPAOs*; green represents negative correlation, and red represents positive correlation.

To generate more clues on the biological roles of the *GmPAOs*, we studied their expression levels in various soybean organs/tissues. Generally speaking, more than half of the group I *GmPAOs* are ubiquitously expressed in soybean, like *GmPAO10* and *GmPAO3*, whose expression was detected in all nine tested samples (Figure 5a). Interestingly, except for *GmPAO4*, whose transcript is more abundant in pod and leaves, the rest of the group I genes all show their highest expression level in flower, strongly implying that they play key functions in soybean flower development. For the group II genes, *GmPAO5* and *GmPAO12* are predominantly expressed, and both show preference in soybean shoot apical meristem (SAM) and stem. In contrast, *GmPAO6* and *GmPAO1* in group III are specifically highly expressed in flower, whereas *GmPAO13* and *GmPAO7* are barely expressed in all samples (Figure 5a). Meanwhile, we also investigated the gene expression correlation of the *GmPAOs* and found that genes in the same group tend more to positively correlate with each other (Figure 5b). *GmPAO6* and *GmPAO1* also show strong positive correlation with group I members, while group II genes were negatively correlated with other *GmPAOs*, hinting that this group has developed specific functions in soybean plants.

**Figure 6:**
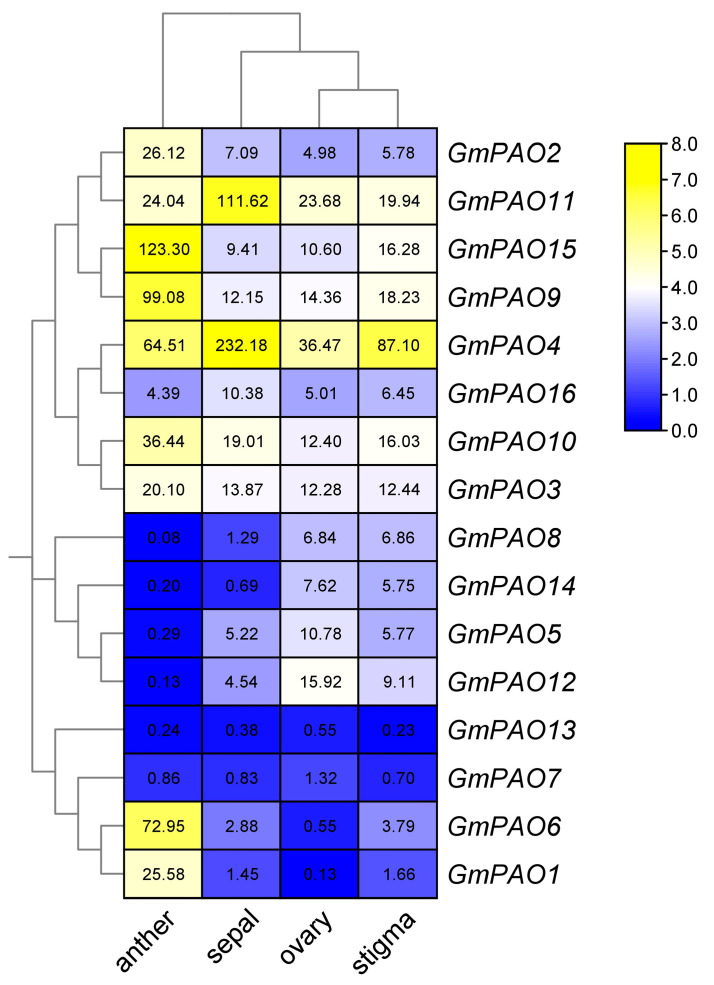
Expression pattern of *GmPAOs* in anther, sepal, ovary, and stigma. Numbers in the color blocks indicate FPKM values. The color scale represents log2 format-converted FPKM values, with yellow indicating high expression levels and blue indicating low expression levels.

Since many *GmPAOs* show preference in soybean flower, we also checked their detailed expression profiles in four floral tissues and found that most of them also displayed tissue specificity (Figure 6). *GmPAO2*, *15*, *9*, *10*, and *3* in group I all have their highest expression in anther. Taking *GmPAO15* as an example, its expression level in anther is 7.6 or more times higher than that in stigma, ovary, and sepal. The other three group I genes, however, are predominantly expressed in sepal. Group II *GmPAOs* are mainly expressed in ovary and stigma, while *GmPAO6* and *GmPAO1* from group III are almost uniquely expressed in anther. These results demonstrate that different *GmPAOs* play major roles in specific floral parts, and they function corporately to regulate soybean flower development.

### 2.8. Expression Profiling of GmPAOs Under Abiotic Stress

**Figure 7:**
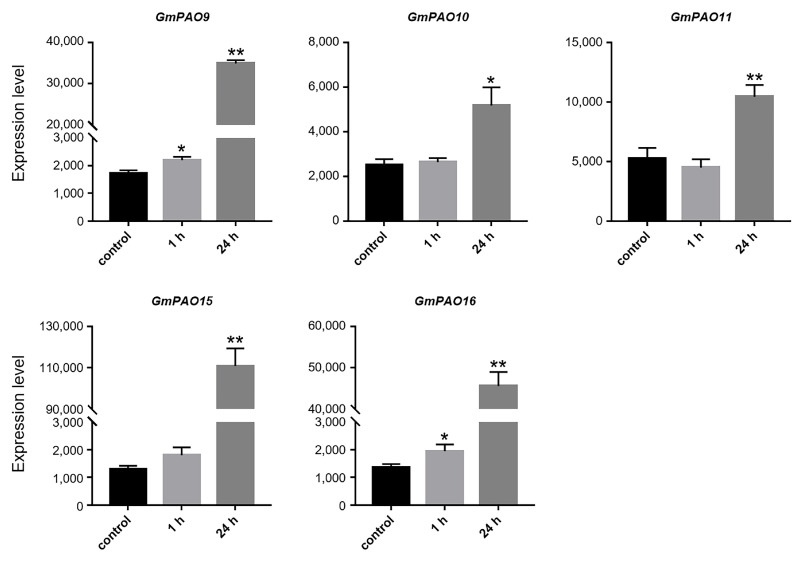
Expression profile of *GmPAOs* in soybean leaves under cold stress. Gene expression level is represented as DESeq-normalized transcript counts. Error bars indicate standard deviations of three replicates. Asterisks stand for significant differences (Student’s *t*-test), * *p* < 0.05, ** *p* < 0.01.

We also analyzed the expression patterns of the *GmPAOs* under stressful environments and found that they were mainly responsive to cold, drought, and submergence treatment. Under cold condition, five genes are significantly induced following 24 h of stress, but their expression levels are basically not affected by 1 h of treatment (Figure 7). Among them, the expression of *GmPAO15* was mostly promoted, which was increased by 85.0 times after cold stress. Those of the remaining four, *GmPAO9*, *10*, *11*, and *16*, were increased by 20.2, 2.1, 2.0, and 33.2 times, respectively. Several other *GmPAOs* were also induced by cold stress, but their expression levels were much lower than those of the above five genes (Table S4).

**Figure 8:**
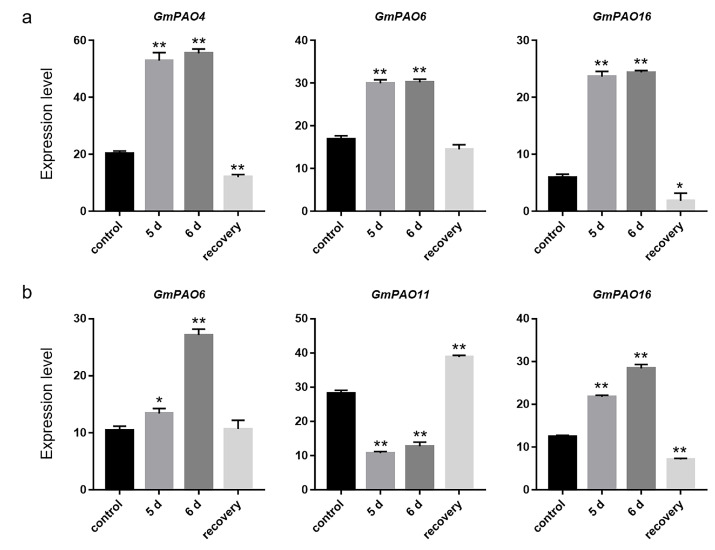
Expression profile of *GmPAOs* in soybean root (**a**) and leaves (**b**) under drought stress. Gene expression level is represented as FPKM values. Error bars indicate standard deviations of three replicates. Asterisks stand for significant differences (Student’s *t*-test), * *p* < 0.05, ** *p* < 0.01.

Under drought conditions, three genes, *GmPAO4*, *6*, and *16*, were enhanced by stress treatment in soybean root (Figure 8a). In leaves, the expression of *GmPAO6* and *GmPAO16* also increased, while that of *GmPAO11* was decreased by drought stress instead (Figure 8b). The other *GmPAOs* were not markedly influenced by drought treatment, either in root or in leaves (Table S5). It is noteworthy that the cold- and drought-responsive *GmPAO* genes all belong to group I, suggesting that this group, rather than the other two, mainly contributed to soybean response to cold and drought stress.

**Figure 9:**
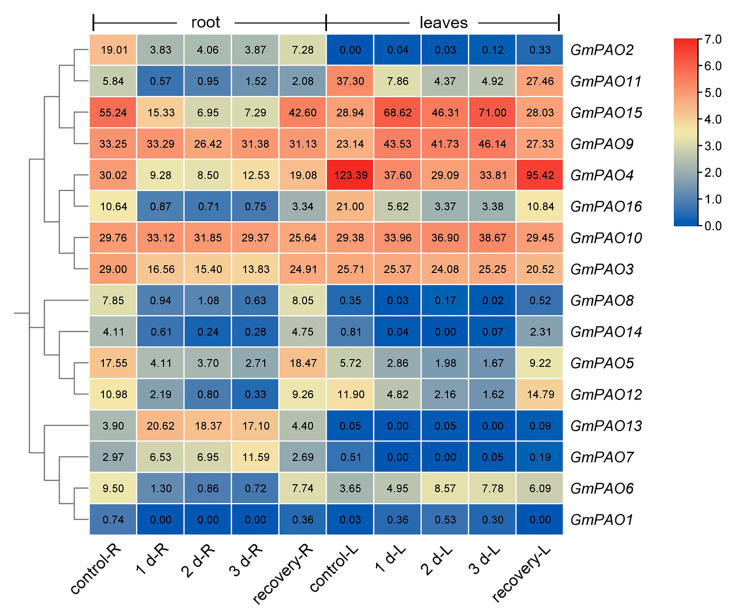
Expression profile of *GmPAOs* in soybean root and leaves under submergence stress. Numbers in the color blocks indicate mean FPKM values of three replicates. The color scale represents log2 format-converted FPKM values, with red indicating high expression levels and blue indicating low expression levels.

The *GmPAOs* seem more vital to soybean response to submergence stress, as nearly all of them were found disturbed under such condition (Figure 9). In root, most *GmPAOs* from group I and II were repressed by submergence treatment, and following recovery, their expression partially or fully returned to initial levels. Only *GmPAO9* and *GmPAO10* remained unchanged throughout the whole process. *GmPAO6* from group III was also repressed by submergence. However, *GmPAO13* and *GmPAO7*, whose expression levels were somewhat low under the control condition, increased by up to 5.3 and 3.9 times, respectively, after stress, and both returned to initial levels following 1 d of recovery. In soybean leaves, less genes were responsive to submergence, as some of them were not expressed in this organ at all (Figure 9), even though approximately half of the *GmPAOs* were either induced or inhibited by the stress treatment. Expression of *GmPAO15*, *9*, and *6* were increased, but that of *GmPAO11*, *4*, *16*, *5*, and *12* was decreased instead. Similarly, following recovery treatment, their expression levels also nearly returned. These results indicate that the *GmPAOs* are crucial to soybean submergence response, both in root and leaves, but certain genes might function diversely in the two organs.

## 3. Discussion

### 3.1. Identification and Evolution of Soybean PAO Gene Family

PA homeostasis, which is crucial to numerous biological pathways of plants, is regulated by a number of anabolic and catabolic enzymes, of which PAO mediates the catabolism of PAs [1]. While the *PAO* genes have been identified in many plant species, little is known about this gene family in soybean. Here, a systematic characterization on soybean *PAOs* is presented.

Genome-wide analysis identified 16 *GmPAOs* from the soybean genome (Table 1), which is much more than the *PAO* homologs in *Arabidopsis* (5), *Brachypodium distachyon* (5), tomato (7), rice (7), and maize (9) [12,26]. A previous report showed that soybean has the largest transcriptome (64.6 MB) among the above-referenced species, and the transciptomes of *Arabidopsis*, tomato, rice, maize, and *Brachypodium distachyon* are 40.2, 37.5, 42.0, 55.5, and 61.5 MB, respectively [36]. Therefore, the transcriptome size partially accounts for the higher number of *PAO* members in soybean than in the others. Additionally, we also identified 17 duplicated *GmPAO* pairs through collinear analysis (Figure 2); contrasting with that, only two were discovered in maize [12], suggesting that gene duplication further contributed to the expansion of the *GmPAO* family.

Phylogenetic analysis split the PAOs of *Arabidopsis*, rice, and soybean into four groups. It is noteworthy that *Arabidopsis* and soybean have members in groups I, II, and III but not IV, and only rice has members in the last group (Figure 1). It was defined that the *Arabidopsis* and rice homologs in groups I, II, and/or III were all BC-type PAOs, while the group IV PAOs were considered as TC-type that were derived from monocot species [13,22,26]. Accordingly, we hypothesize that all GmPAOs function in the back conversion of PAs, which should be enzymatically confirmed in further studies. Despite the potential similarities in enzymatic activity, the different GmPAO groups exhibited apparent distinctions in terms of gene structure and conserved motif (Figure 3). Genes in group I and III include eight or nine introns, while those in group II are intron-less. This characteristic seems quite conserved across plant species according to the exon–intron analysis of *PAOs* from *Arabidopsis*, maize, rice, potato (*Solanum tuberosum*), and tomato [2,12,37]. The resemblance in gene structure of *PAOs* from various plant species is indicative of their functional conservation during the evolution process, which provides important clues for determining the biological roles of *GmPAOs* in the future.

### 3.2. Role of GmPAOs in Soybean Development and Phytohormone Response

Differentiation of the *PAOs* from the various groups is also reflected in the perspective of tissue-specific expression patterns. In soybean, we observed that most *GmPAOs* in group I were expressed in all tested samples (Figure 5). Consistently, ubiquitous expression of genes in this group was also reported in tomato, pepper, and peach (*Prunus persica)* [26,37,38], suggesting that these homologs might play extensive roles in regulating plant growth and development. Tissue-specific expression of *PAOs* also exists in different plant species. *AtPAO5* in group II, for instance, was mostly expressed in the vascular system of roots and hypocotyls [39], and its close homolog in peach, *PpePAO4*, had high expression in leaves [38]. In soybean, *GmPAO5* and *GmPAO12* of group II showed preference in SAM and stem (Figure 5), implying their involvements mainly in vegetative growth. In comparison, two group III genes, *GmPAO1* and *GmPAO6*, were specifically expressed in flower and, particularly, in anther (Figure 5 and Figure 6), similar to its close homologs in pepper and peach [26,38]. Moreover, the implication of plant *PAOs* in fruit ripening was also reported [37,38]. So jointly, *GmPAOs* are supposed to be responsible for both vegetative and reproductive development in soybean, but how soybean integrates different *GmPAOs* to mediate these processes remains to be clarified.

By analyzing the promoters of the *GmPAOs*, we found that plant hormone-responsive *cis*-acting elements were mostly enriched (Figure 4). Currently, the involvement of plant *PAOs* in phytohormone response has been mainly researched in the model plants *Arabidopsis* and rice. For example, *AtPAO5* is upregulated by cytokinin and functions in the interplay between auxin and cytokinin [17]. Extensive expression profiling analyses indicated that different *AtPAOs* and/or *OsPAOs* were also responsive to IAA, ABA, GA, JA, and SA treatments [40,41]. A recent in-depth study reported that MYC2, the core transcription factor in the JA signaling pathway, could directly bind to the *OsPAO6* promoter to mediate PA catabolism under herbivore attacks [42]. PAs, together with other phytohormones (auxin, cytokinin, ABA, brassinosteroids, strigolactones, gibberellins, MeJA, etc.), were considered important bioregulators to participate in plant development and stress response [43,44]. The identification of different phytohormone-responsive elements in the *GmPAOs*’ promoters enlightened further studies on the interaction between PAs and various plant hormones.

### 3.3. Role of GmPAOs in Response to Cold, Drought, and Submergence Stress

The exogenous application of PAs could boost plants’ broad-spectrum tolerance to both abiotic and biotic stresses, such as drought, salinity, low/high temperature, and pathogenic/bacterial infections [45,46]. Meanwhile, endogenous PA levels were also found to increase in plants and exert protective roles on plants when they are subjected to abiotic stresses [1]. Thus, transcriptional alteration of genes responsible for PA metabolism, including *PAOs*, was reasonably thought to regulate the plant response to stress conditions. Most *SlPAOs* of tomato were induced by cold treatment [37], while the barley *HvPAOs* were repressed [47], and there exist both upregulated and downregulated *PAOs* in rice and *Camellia sinensis* [33,40], suggestive of the complex role of *PAOs* in different plant species. We discovered that five group I *GmPAOs* were remarkably upregulated in response to cold stress (Figure 7), similar to their homologs (*CaPAO2* and *CaPAO4*) in pepper, overexpression of which increased Put, Spd, and Spm contents in *Arabidopsis* and improved cold tolerance by enhancing the antioxidant system and activating cold-responsive genes [26]. This supported an inspiration for research on the functional mechanisms of *GmPAOs* under cold stress.

The role of plant PAOs under drought stress can be opposite among different species. Reduced PAO activity in *Arabidopsis* could enhance drought tolerance by decreasing ROS production and increasing defense gene expression [48]. Furthermore, mutation of tomato *SlPAO3* also improved the tolerance to drought [30]. In maize, however, gene expression and enzyme activity of PAOs in leaves were elevated by drought treatment in both tolerant and sensitive genotypes, but a more prominent enhancement was observed in the former than in the latter [49], implying the positive role of PAOs in maize drought resistance. A later study revealed that *ZmPAOs* might be differently regulated by drought in various maize organs. For example, expression of *ZmPAO6* was induced in root but inhibited in leaves after drought treatment [12]. This is also the case for certain *GmPAOs*, as there are genes specifically up-/downregulated in soybean leaves or roots (Figure 8). We propose that the tissue-specific expression profile of *PAO* genes under drought stress should be taken into account when determining their functional roles under such conditions.

Although there are studies demonstrating that exogenous application or endogenous elevation of PAs could facilitate plant tolerance to submergence/waterlogging conditions [50,51,52,53], how PA metabolism-related genes participate in response to such stress is unknown so far. It was recently reported that the (Spd + Spm)/Put ratio is an important indicator of plant tolerance to waterlogging, and the flood-tolerant peony (*Paeonia lactiflora*) variety had a higher ratio than the intolerant variety when the plants were subjected to waterlogging stress [54]. Considering the role of PAOs in oxidizing Spm/Tspm to Spd and/or Spd to Put, downregulation of the majority of *GmPAOs* under submergence stress might help in maintaining a higher (Spd + Spm)/Put ratio and, thus, further alleviating submergence-induced damages (Figure 9). It is also interesting to note that, although *GmPAO7* and *GmPAO13* were barely expressed in all soybean organs/tissues in most cases, their expression levels were apparently induced in response to submergence (Figure 5 and Figure 9), suggesting that some of the *GmPAOs* function as back-up genes to mediate PA homeostasis under severe environments.

The transcriptome data-based expression analysis of the *GmPAOs* indicated their flower preference and responsiveness to cold, drought, and, especially, submergence stress. However, experimental validations are required to reveal the detailed expression profiles of these genes, and further analysis of mutants and overexpressors of *GmPAOs* will help better expound their functional roles in soybean development and abiotic stress response.

## 4. Materials and Methods

### 4.1. Identification of GmPAOs from Soybean Genome and Physiochemical Analysis

The amino acid sequences of *Arabidopsis* and rice PAO proteins were retrieved from the Phytozome database (https://phytozome-next.jgi.doe.gov/, accessed on 19 November 2023), which were then used as query sequences to conduct a local BLASTP search against soybean genome data, with an e-value of 1 × 10^−5^ (*G. max* Wm82.a2.v1, acquired from Phytozome). The InterPro database (https://www.ebi.ac.uk/interpro/, accessed on 19 November 2023) was used to confirm the amino oxidase domain (PF01593) and remove redundant/short sequences. The remaining candidates were considered GmPAOs, whose protein sequences were downloaded from Phytozome and then input to the ExPasy ProtParam tool (https://web.expasy.org/protparam/, accessed on 19 November 2023) to compute their molecular weight (MW), isoelectric point (pI), instability index, and grand average of hydropathicity (GRAVY).

### 4.2. Chromosomal Mapping and Phylogenetic Analysis

The soybean genome annotation file was downloaded from the Phytozome database, and the ID numbers of the *GmPAOs* were loaded into TBtools (v2.112) to map all genes to soybean chromosomes [55]. Phylogenetic analysis was carried out by MEGA-X software. Firstly, the amino acids of PAO proteins from soybean, *Arabidopsis*, and rice were imported to MEGA-X to perform multiple alignment using the ClastalW algorithm; then, an evolutionary tree was constructed through the maximum likelihood method and Le_Gascuel_2008 model [56]. The reliability of the phylogenetic trees was tested by bootstrap analysis with 1000 replicates.

### 4.3. Analysis of Collinear Relationship and Ka/Ks Values

The genome data of soybean were loaded into TBtools (v2.112) to perform a collinear analysis, and the Circos tool was employed to visualize the collinear relationship of the *GmPAOs*. Non-synonymous substitution rate (Ka) and synonymous substitution rate (Ks) of duplicated gene pairs were computed with the Ka/Ks Calculator, and the selective pressure was evaluated based on the Ka/Ks ratios (>1 represents positive selection, =1 represents neutral selection, and <1 represents negative selection). The Ks values were further utilized to calculate the divergence time of the duplicated gene pairs according to the following formula: T = Ks/2λ. The synonymous mutation rate (λ) for soybean is 6.1 × 10^−9^ substitutions per synonymous site per year [57].

### 4.4. Analysis of Conserved Motifs, Gene Structure, Protein Structure, and Cis-Acting Elements

The amino acid sequences of the GmPAOs were loaded into MEME suite (https://meme-suite.org/meme/, accessed on 19 November 2024) to analyze their conserved motifs. TBtools software (v2.112) was used to visualize the exon–intron structure of the *GmPAO* genes based on the genome annotation file of soybean. The putative secondary structures of the GmPAOs were predicted by SOPMA (https://npsa-prabi.ibcp.fr/cgi-bin/npsa_automat.pl?page=npsa%20_sopma.html, accessed on 23 December 2024), and the AlphaFold server (https://alphafold.ebi.ac.uk/, accessed on 24 December 2024) was hired to produce their tertiary models. The 2000 bp DNA sequence upstream of the gene start codon was analyzed by the PlantCARE database (http://bioinformatics.psb.ugent.be/webtools/plantcare/html/, accessed on 24 December 2024) to investigate the *cis*-acting elements in the promoters of the *GmPAOs*.

### 4.5. Expression Analysis of GmPAOs in Different Organs and Floral Tissues

To analyze the expression pattern of the *GmPAOs* in different soybean organs, the RNA abundance in root, root hairs, nodules, stem, leaves, shoot apical meristem (SAM), flower, pod, and seed, represented by fragments per kilobase of exon per million mapped fragments (FPKM), were searched in the Phytozome database, based on which clustering heatmap was produced with TBtools software. Then, a correlation heatmap was generated using the Expression Correlation Calc plugin [55].

To further analyze the expression level of the *GmPAOs* in flower, transcriptome data in four flower parts (anther, sepal, ovary, and stigma) of R1 stage soybean plants (GSE218146), were obtained from the NCBI GEO repository (https://www.ncbi.nlm.nih.gov/geo/, accessed on 12 November 2022) and were then used to create a clustering heatmap.

### 4.6. Expression Profiling Under Abiotic Stress

The RNA-seq data of soybean (GSE117686) were downloaded from the GEO repository to analyze the expression pattern of the *GmPAOs* under cold stress. To conduct cold treatment, soybean seedlings of two weeks old were subjected to 4 °C condition. Plants grown at 22 °C were used as the control. The unifoliate leaves were sampled at 0, 1, and 24 h to perform transcriptome sequencing [58]. The expression data of the *GmPAOs* in response to drought and submergence stress were acquired from the Plant Public RNA-seq Database (https://plantrnadb.com/, accessed on 15 November 2024) to study their expression profiles under such conditions. For drought treatment, soybean plants at the V1 stage were dehydrated for 5 or 6 d and were recovered for 1 d after 6 d of drought; plants grown under irrigated conditions were used as the control. Submergence stress was conducted as follows: V1 plants were submerged in water for 1, 2, or 3 d and recovered for 1 d after 3 d of stress; plants before submergence were used as the control. Leaf blades and roots of the above materials were sampled to conduct transcriptome analysis [59].

## 5. Conclusions

The current study identified 16 *GmPAOs* from the soybean genome, which were distributed in nine chromosomes and were phylogenetically classified into three groups. Collinear analysis indicated that gene duplication contributed largely to the expansion of the family, and different members were found to exhibit similarities and differences in terms of conserved motif, gene structure, and protein structures. Promoter investigation suggested that different *GmPAOs* might participate in multiple developmental, phytohormone responsive, and stress responsive pathways. *GmPAO* genes exhibited various tissue expression patterns, with some being ubiquitously expressed and some being tissue specific. Finally, expression profiling revealed that the *GmPAOs* were involved in the response to cold, drought, and, especially, submergence stress. These results lay an important foundation for further functional excavation of *GmPAOs* in soybean.

## Figures and Tables

**Table 1:** Sequence information and physiochemical parameters of soybean polyamine oxidases.

Gene Name	Gene ID	DNA (bp)	Transcript (bp)	Peptide (aa)	MW (kDa)	pI	Instability Index	GRAVY
*GmPAO1*	Glyma.02G018800	5022	1158	385	43.29	5.60	38.08	−0.181
*GmPAO2*	Glyma.02G240000	7474	2073	494	54.90	5.81	35.44	−0.026
*GmPAO3*	Glyma.02G282000	4303	2434	487	54.11	5.68	37.47	−0.062
*GmPAO4*	Glyma.08G303800	5622	2087	490	54.15	5.59	36.28	−0.077
*GmPAO5*	Glyma.09G063000	2834	2834	600	65.91	5.82	43.26	−0.339
*GmPAO6*	Glyma.09G227500	7608	2516	465	52.18	5.18	39.87	−0.274
*GmPAO7*	Glyma.10G090700	8933	2086	500	56.47	5.00	44.91	−0.267
*GmPAO8*	Glyma.13G104100	1617	1617	538	59.38	6.06	42.81	−0.222
*GmPAO9*	Glyma.13G153100	6460	2550	493	54.44	5.59	28.01	−0.002
*GmPAO10*	Glyma.14G032300	4385	2413	489	54.34	5.78	36.58	−0.058
*GmPAO11*	Glyma.14G209400	7760	2268	494	54.65	5.60	37.04	−0.031
*GmPAO12*	Glyma.15G169600	3473	2969	581	63.67	6.00	39.77	−0.312
*GmPAO13*	Glyma.15G276500	9075	2063	501	56.17	5.20	40.19	−0.262
*GmPAO14*	Glyma.17G055000	2346	2346	530	58.31	5.93	42.27	−0.200
*GmPAO15*	Glyma.18G045100	6641	2597	493	54.50	5.79	28.32	−0.049
*GmPAO16*	Glyma.18G116300	4266	1861	498	55.05	5.79	34.69	0.105

MW: molecular weight; pI: isoelectric point; GRAVY: grand average of hydropathicity.

## Data Availability

The original data presented in this study are included in the article and Supplementary Materials. Further inquiries can be directed to the corresponding author.

## Supplementary Materials

The following supporting information can be downloaded at: https://www.mdpi.com/article/10.3390/plants14121867/s1, Figure S1: Chromosomal localization of *GmPAOs*. Figure S2 Sequence details of the 6 conserved motifs. Figure S3: Tertiary structure of GmPAOs. Table S1: Ka/Ks values and divergence time of *GmPAO* gene pairs. Table S2: Prediction of the secondary structure of GmPAOs. Table S3: The *cis*-acting elements in promoters of *GmPAOs*. Table S4: Expression data of *GmPAOs* under cold stress. Table S5: Expression data of *GmPAOs* under drought stress.
