# Supplementary material for: Genome-Wide Analysis of Soybean Polyamine Oxidase Genes Reveals Their Roles in Flower Development and Response to Abiotic Stress
Source: Plants (Basel). 2025 Jun 18;14(12):1867. doi: 10.3390/plants14121867 (PMC12196569; doi:10.3390/plants14121867)
Supplement: Supplementary file 1 [file plants-14-01867-s001.zip › Table S1.pdf]

Table S1. Ka/Ks values and divergence time of *GmPAO* gene pairs.

Gene 1	Gene 2	Ka	Ks	Ka/Ks	Purifying selection	Divergence time (mya)
GmPAO2	*GmPAO11*	0.0278	0.1259	0.2208	Yes	10.3197
GmPAO2	*GmPAO15*	0.1138	0.5112	0.2226	Yes	41.9016
GmPAO3	*GmPAO4*	0.0954	0.5887	0.1621	Yes	48.2541
GmPAO3	*GmPAO10*	0.0153	0.1013	0.1510	Yes	8.3033
GmPAO3	*GmPAO16*	0.0990	0.5553	0.1783	Yes	45.5164
GmPAO4	*GmPAO10*	0.0914	0.5597	0.1633	Yes	45.8770
GmPAO4	*GmPAO16*	0.0499	0.1458	0.3422	Yes	11.9508
GmPAO5	*GmPAO8*	0.1380	0.5445	0.2534	Yes	44.6311
GmPAO5	*GmPAO12*	0.0379	0.1279	0.2963	Yes	10.4836
GmPAO5	*GmPAO14*	0.1363	0.5811	0.2346	Yes	47.6311
GmPAO7	*GmPAO13*	0.0360	0.1732	0.2079	Yes	14.1967
GmPAO8	*GmPAO12*	0.1308	0.5437	0.2406	Yes	44.5656
GmPAO8	*GmPAO14*	0.0228	0.1052	0.2167	Yes	8.6230
GmPAO9	*GmPAO15*	0.0184	0.0922	0.1996	Yes	7.5574
GmPAO10	*GmPAO16*	0.0958	0.5279	0.1815	Yes	43.2705
GmPAO11	*GmPAO15*	0.1274	0.4740	0.2688	Yes	38.8525
GmPAO12	*GmPAO14*	0.1313	0.5492	0.2391	Yes	45.0164
