# Supplementary material for: Genome-Wide Analysis of Soybean Polyamine Oxidase Genes Reveals Their Roles in Flower Development and Response to Abiotic Stress
Source: Plants (Basel). 2025 Jun 18;14(12):1867. doi: 10.3390/plants14121867 (PMC12196569; doi:10.3390/plants14121867)
Supplement: Supplementary file 1 [file plants-14-01867-s001.zip › Table S2.pdf]

Table S2. Prediction of the secondary structure of GmPAOs.

Protein	α-helix	extended strand	random coil
GmPAO1	31.69%	20.00%	48.31%
GmPAO2	36.64%	17.21%	46.15%
GmPAO3	39.43%	15.61%	44.97%
GmPAO4	38.78%	16.94%	44.29%
GmPAO5	34.83%	14.83%	50.33%
GmPAO6	33.33%	18.92%	47.74%
GmPAO7	36.00%	16.00%	48.00%
GmPAO8	35.87%	16.54%	47.58%
GmPAO9	36.51%	16.84%	46.65%
GmPAO10	36.61%	16.97%	46.42%
GmPAO11	36.64%	17.41%	45.95%
GmPAO12	33.91%	14.63%	51.46%
GmPAO13	34.93%	18.16%	46.91%
GmPAO14	34.15%	15.85%	50.00%
GmPAO15	36.51%	16.23%	47.26%
GmPAO16	39.96%	17.67%	42.37%
