# Supplementary figures and images for: Genome-Wide Analysis of Soybean Polyamine Oxidase Genes Reveals Their Roles in Flower Development and Response to Abiotic Stress
Source: Plants (Basel). 2025 Jun 18;14(12):1867. doi: 10.3390/plants14121867 (PMC12196569; doi:10.3390/plants14121867)

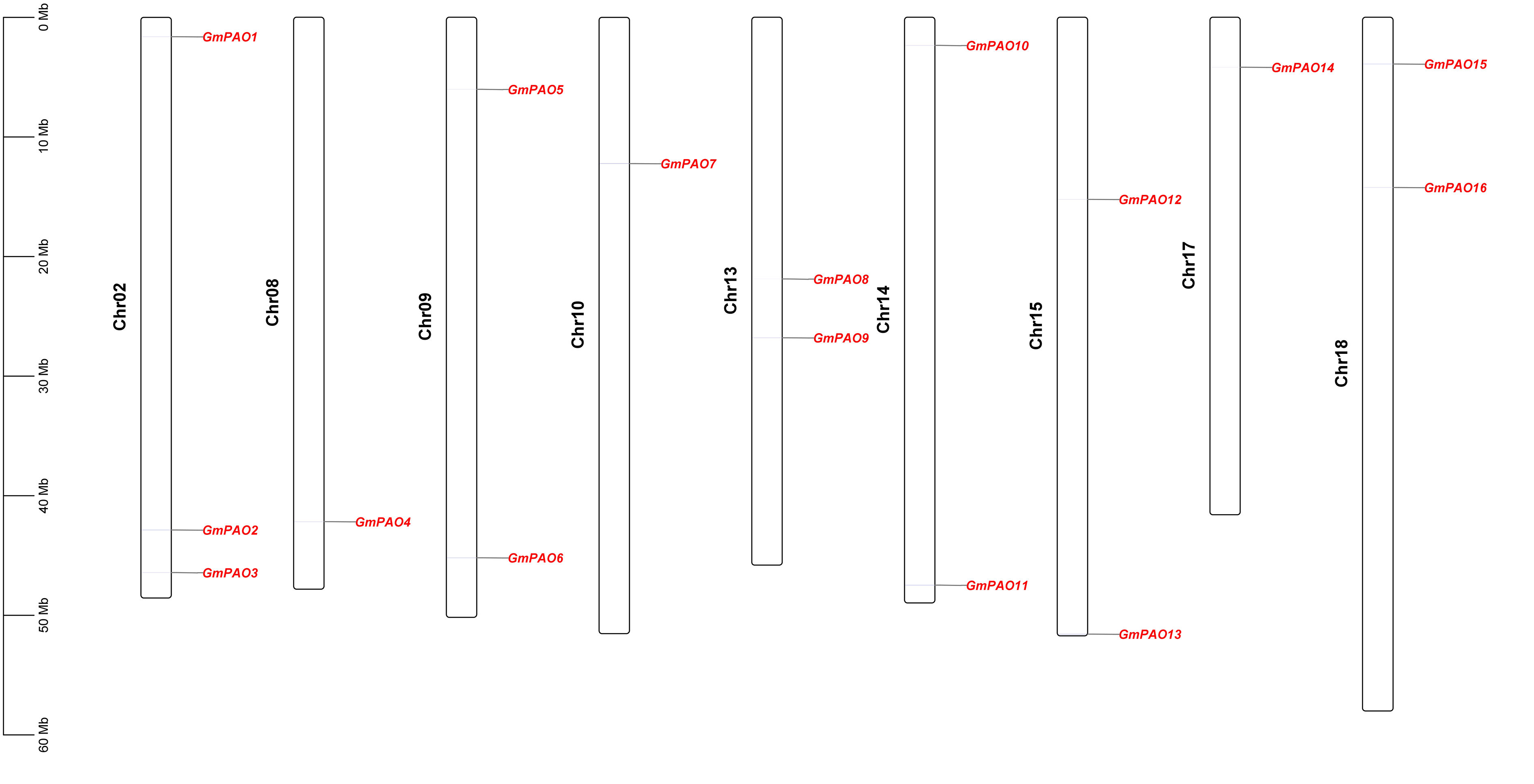

Supplement: Supplementary file 1 [file plants-14-01867-s001.zip › Figure S1.tif]

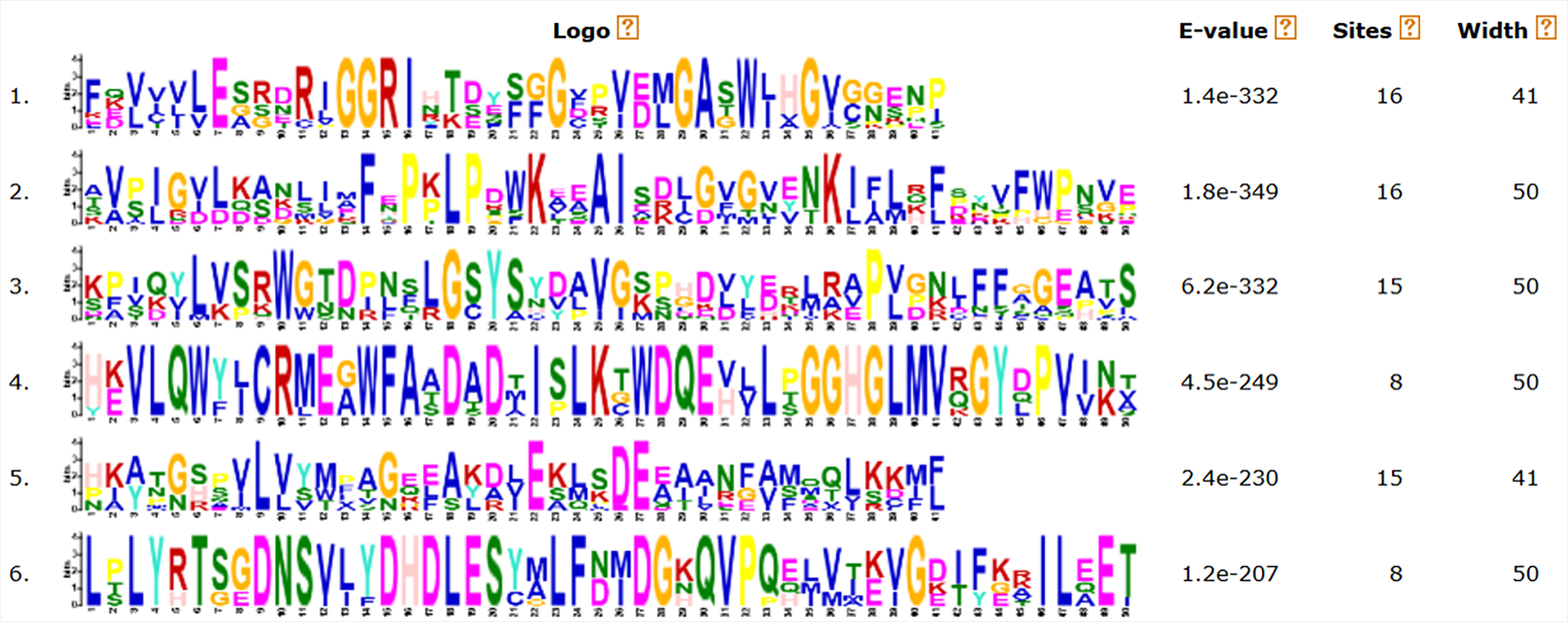

Supplement: Supplementary file 1 [file plants-14-01867-s001.zip › Figure S2.tif]

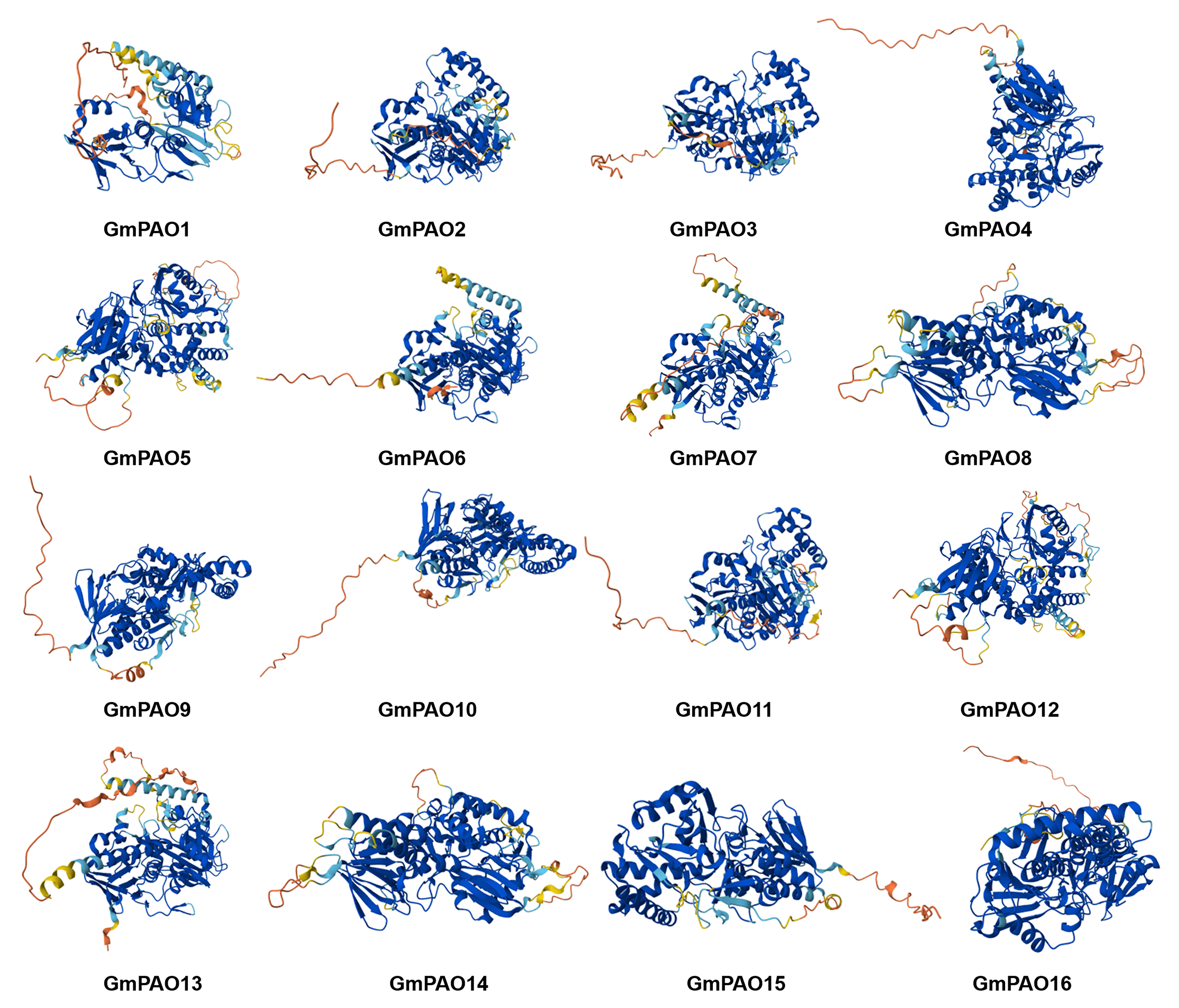

Supplement: Supplementary file 1 [file plants-14-01867-s001.zip › Figure S3.tif]
